# Lived experiences of farmworkers from five U. S. states during the COVID-19 pandemic

**DOI:** 10.3389/fpubh.2025.1503383

**Published:** 2025-06-10

**Authors:** Keren Herrán, Nicandro Mandujano-Acevedo, Jocelyn Claudel Suarez, Bethany Boggess, Edward A. Frongillo

**Affiliations:** ^1^Department of Health Promotion, Education, and Behavior, Arnold School of Public Health, University of South Carolina, Columbia, SC, United States; ^2^National Center for Farmworker Health, Buda, TX, United States

**Keywords:** farmworker, COVID-19, qualitative methods, vaccine, family, workplace

## Abstract

**Objective:**

The nexus of farmworker and COVID-19 peer-reviewed research has yet to be advanced by qualitative analysis that: (1) focuses on multiple dimensions of farmworker’s daily life and (2) uses a geographically diverse sample. The present data collection project fills this gap by using the National Center for Farmworker Health’s (NCFH) Farmworker COVID-19 Community Assessment (FCCA) Phase 2 dataset which contains a varied sample of farmworkers and local experts across selected counties in five states. The NCFH FCCA Phase 2 data were analyzed to characterize how farmworkers from a multistate sample experienced COVID-19 impact their daily lives, with particular focus on understanding farmworker vaccine experiences, familial dynamics, and actions implemented by employers.

**Methods:**

Participants (*n* = 28; farmworker n_1_ = 19, local expert n_2_ = 9) were recruited via purposive and snowball sampling. NVivo software and grounded theory coding were used for data analysis. Techniques utilized to ensure rigorous qualitative research were: (1) continuously applying researcher reflexivity, (2) purposive sampling, and (3) investigator and data triangulation.

**Results:**

COVID-19 primarily impacted three spheres of farmworker’s daily life: health, family, and the workplace. Of the emergent themes, novel findings include farmworkers’ motivation for vaccination, farmworkers’ anguish concerning extended family, the deterioration of unity in farmworkers’ families, and identification of what workplace changes farmworkers deemed helpful. These novel findings widen understanding of how farmworker health can be promoted in the event of another pandemic. Although present recommended strategies (e.g., improving housing conditions and disseminating health information in Spanish) are valuable in ensuring optimal well-being of farmworkers long-term, policymakers and public health professionals should also design and integrate actions that target farmworkers’ vaccine motivations, promote unity/connection within the extended and nuclear family, and incentivize employers to implement workplace changes that farmworkers value.

## 1 Introduction

Over 2 million migrant and seasonal farmworkers labor in the United States and are essential for the nation’s $1.053 trillion food and agriculture industry (1, 2). Despite farmworkers’ vital contributions to the U. S. food supply and economy, farmworkers face several socioeconomic and health disparities. Agriculture has the highest incidence of fatal workplace injuries of all industries (3). Occupational hazards include use of heavy machinery, intense physical labor, and pesticide exposure. Contact with toxic chemicals used in farmwork can cause long-term consequences such as infertility, neurological disorders, and cancer (4). Further compounding these health risks, 82% of farmworkers lack comprehensive employer-provided health insurance, 44% of employer-provided manufactured housing for farmworkers is moderately or severely inadequate, 42% of farmworkers are undocumented, and 21% of farmworkers live below the poverty line due to low wages and wage theft (5–8). Understanding such social determinants of health is important since these determinants precede and shape individual health behavior, exposure to health hazards, and access to supportive health resources (9).

For vulnerable populations, intersectionality of inequities may exacerbate their susceptibility to disease and its consequences (10, 11). Multiple marginalized U. S. populations, such as farmworkers, were disproportionately affected by the COVID-19 pandemic (12). The National Center for Farmworker Health (NCFH) in collaboration with the Centers for Disease Control and Prevention (CDC) developed data-collection methodologies (i.e., Farmworker COVID-19 Community Assessments -FCCAs) to assess farmworkers’ COVID-19 knowledge, attitudes, practices, access to vaccination and testing, and develop actionable evidence-based strategies based on data from representative samples of farmworkers. FCCA Phase 1 data were collected from August to December of 2021 in California, Texas, New Mexico, Mississippi, and Florida. The interview guides used to collect FCCA data were refined after collection of FCCA Phase 1 data to match the changing conditions of the COVID-19 pandemic. FCCA Phase 2 data were collected from March to August of 2022 in selected counties in Georgia, North Carolina, Colorado, New Jersey, and Washington. Analysis of these data will be valuable for application to future disease outbreaks or pandemics that may render similar unfavorable health contexts for farmworkers.

Existent literature on the lived experiences of farmworkers during the pandemic is specific to particular regions and crop productions or focuses solely on a specific dimension of farmworkers’ lives, such as worker compensation coverage. For instance, Handal et al. studied the workplace and living conditions among farmworkers in Michigan during COVID-19, Keeney et al. evaluated the mental health stressors among Latina female farmworkers in Imperial County, California, and Gehlbach et al. reported on COVID-19 testing and vaccine hesitancy among Latino farmworkers in East Coachella Valley, California (13–15). The nexus of farmworker and COVID-19 peer-reviewed research has yet to be advanced by qualitative analysis that: (1) focuses on multiple dimensions of farmworker’s daily life and (2) uses a geographically diverse sample. We filled this gap by using the NCFH FCCA Phase 2 dataset which contains a varied sample of farmworkers and local experts from six counties in five states and can capture the nuances in lived experiences among farmworkers nationwide during the pandemic. The NCFH FCCA Phase 2 data were analyzed to characterize how farmworkers from a national sample experienced COVID-19 impact their daily lives, with particular focus on understanding farmworker vaccine experiences, familial dynamics, and actions implemented by employers.

## 2 Methods

### 2.1 Research team and reflexivity

NCFH is a private not-for-profit corporation committed to improving the health of farmworker families. NCFH provides data services, training and technical assistance, and various products to health centers, universities, researchers, and individuals engaged in farmworker health. During COVID-19, NCFH produced COVID-19 educational material, managed a healthcare referral services hotline, and provided COVID-19 prevention training to agricultural employers, among other services. NCFH’s active involvement in promoting farmworker health before and during the pandemic established rapport and trust with research participants. Participants were forthright and detailed in their response to interview questions, recognizing that NCFH valued their firsthand insight.

Interviews were conducted by NCFH Research and Evaluation team members trained in qualitative data collection methods. Interviewers were fluent in Spanish and had prior experience in facilitating interviews and engaging with vulnerable populations. Team members collaborated in creating the interview guide with CDC staff, and rehearsed interviewing techniques prior to conducting interviews. Feedback from participant interviews in NCFH FCCA Phase 1 enhanced the quality and clarity of questions asked in NCFH FCCA Phase 2 data collection.

### 2.2 Participant eligibility and sampling

Farmworkers were included if they were employed or had been employed in crop and animal production and support activities (North American Industry Classification System codes 111, 112, 1,151, or 1,152) one day or more since March 15, 2020, in one of the selected counties. The rationale for this inclusion criterion was to capture the experiences of all who labored as a farmworker at some point after the pandemic shutdowns began. Local expert participant inclusion criteria were: (1) involvement in providing support services to farmworkers (e.g., community health workers or legal aid workers), (2) having helped farmworkers one day or more since March 15, 2020, or (3) being a local farmworker leader in their community in one of the selected counties.

Interviews were conducted in Colquitt County, Georgia; Sampson County, North Carolina; Weld County, Colorado; Atlantic and Cumberland counties, New Jersey; and Yakima County, Washington (Tables 1, 2). These interview locations were chosen based on geographic diversity; having more than an estimated 1,000 farmworkers employed in the county and having a significant or growing number of H-2A guest workers employed in the county. According to NCFH estimates, there were 89,369 farmworkers in the counties sampled, representing approximately 4.06% of the 2.2 million farmworkers employed nationally on an annual basis (2). Although California has a large proportion of farmworkers, due to time, logistics, and resources, inclusion of farmworkers in California was not possible at the time of the data collection project.

**Table 1:** Farmworker sample characteristics (*n* = 19).

State where employed	Country of origin	Gender	Specific crop or livestock of specialty during the pandemic	Years of experience working as a farmworker in the U. S.	Vaccination status	COVID-19 infection history	Marital status	Parental status	Legal status	Indigenous languages spoken	Living situation
CO = 5 GA = 1 NC = 5 NJ = 4 WA = 4	El Salvador = 1 Mexico = 17 Puerto Rico = 1	Female = 7 Male = 12	Blueberry = 1 Broccoli = 1 Cabbage = 1 Dairy farm = 6 Flower = 4 Hops = 1 Melon = 1 Peach = 1 Peas = 1 Sweet potato = 2 Tobacco = 4 Tomato = 1	<1 = 4 1 = 2 2–8 = 4 13–18 = 3 20–25 = 4	Vaccinated = 15 Unvaccinated = 4 Booster vaccine obtained = 6	Contracted COVID-19 at least once = 6 Never contracted COVID-19 = 13	Married = 11 Single = 5 Widow = 1	Is a parent = 13 Is not a parent = 5	H-2A = 2 Undoc-umented = 3 U. S. citizen = 1	Nahuatl = 1 Triqui = 1 Tseltal = 1	Lives in employer-provided housing = 4 Lives with nuclear family = 8 Shares a living space with co-workers = 6
			^X^ = 2	^X^ = 2			^X^ = 2	^X^ = 1	^X^ = 13		^X^ = 1

^X^The number of farmworkers for which a characteristic’s information is not disclosed.

**Table 2:** Local expert sample characteristics (*n* = 9).

State where employed	Occupation^X^	Years of experience working with farmworkers
CO = 1	Advocate/organizer for farmworkers = 1	2 = 6
GA = 3	Health outreach services coordinator for farmworkers = 2	15 = 2
NC = 1	Legal services provider for farmworkers = 2	40 = 1
NJ = 2	Support services information expert for farmworkers = 2	
WA = 2	School migrant case manager = 1 College assistance coordinator for farmworker family college students = 1 Community navigator and organizer at community-based organization = 1	

^X^One local expert had two distinct occupational roles serving farmworkers, therefore the total for this column does not match the sample number.

NCFH Research and Evaluation team members partnered with local community health workers, outreach workers, and community members, to conduct in-person quantitative surveys on farmworkers’ vaccination coverage, structural barriers to vaccination, and their COVID-19, knowledge, attitudes and practices (these surveys are not included in this publication). Community health workers, outreach workers, and community members who helped with participant recruitment were contracted by NCFH and compensated for their time. Participants were recruited via purposive and snowball sampling. Local experts were recruited via recommendations from local stakeholders, internet searches concerning farmworker-serving organizations in the area, and/or referral from other local experts. Farmworkers were recruited from quantitative survey participants or by referral from community health workers and outreach workers. NCFH local outreach community health worker partners helped identify these locations to ensure a diverse group of farmworkers were sampled. In-depth individual interviews are an effective method for gathering detailed information through open-ended questions. A total of 40 interviews were conducted with farmworkers and local experts; no one who agreed to be interviewed subsequently declined. For the purposes of this qualitative analysis, saturation was reached after analyzing 19 farmworker and 9 local expert interviews from the Phase 2 dataset. About 9–16 interviews are sufficient to reach data saturation for heterogeneous samples (16).

### 2.3 Data collection

Verbal informed consent from each participant was obtained by NCFH staff members before interviews. Interviewees were explained the purpose of the data collection project, their right to skip questions or terminate the interview at any time, and opportunity to ask clarifying questions. Data were collected via audio recording of interviews. The local experts were interviewed via video call. For farmworkers, interviews were either conducted via video call or in the privacy of their residence. An interviewer and a note taker were present at each interview. A Tseltal interpreter was present for facilitation of one of the interviews. Interviews were in Spanish or English and ranged from 30 to 60 min. Farmworkers and local experts were paid $100 for their participation. Data were collected from March to August of 2022. Additional details on FCCA data collection design can be read in NCFH technical reports (17). This data collection was reviewed by CDC and conducted consistent with applicable federal law and CDC policy (18).

Both the farmworker and local expert in-depth interview guides consisted of main, follow-up, contingent follow-up, and probe questions (Appendices 1 and 2). The farmworker interview guide’s introductory questions were on the interviewee’s job and occupational tasks. Next, interview questions centered on farmworkers’ labor experiences during the pandemic, such as what work challenges arose due to COVID-19 and what occurred if the participant or a co-worker could not work due to infection. The third interview section focused on vaccination experiences, including understanding vaccination barriers and vaccine booster uptake experiences. The last two sections of questions evaluated how COVID-19 affected daily life and identified what issues emerged or persisted during the pandemic. Farmworker interviews ended with a demographic questionnaire.

The local expert interview guide’s introductory question was on the interviewee’s occupation. Subsequently, interview questions centered on characterizing the demographics and migration patterns of the farmworkers the interviewee serves. The third interview section focused on COVID-19 vaccination and COVID-19 services access, including understanding what actions facilitated farmworker vaccination and what challenges farmworkers faced in accessing COVID-19 services such as quarantine housing. The last three sections of questions assessed COVID-19’s impact on farmworkers’ employment and daily life, emerging or current issues in the farmworker community, and farmworkers’ access to general health services. The local expert interviews ended with a demographic questionnaire.

The FCCA Phase 2 farmworker sample includes laborers from distinct Latin American countries, living in different states across the U. S., and working with a myriad of livestock and crops. The sample also includes female, seasonal, migratory, undocumented, H-2A, experienced, novice (individuals with less than 3 years working in the U. S.), and indigenous farmworkers. The NCFH FCCA Phase 2 dataset also includes interviews with local experts (e.g., community health workers and farmworker advocates and leaders).

### 2.4 Data analysis

NVivo software (NVivo 14, Lumivero, Denver, CO) was used for both audio to text transcription and coding. Data analysis was conducted from November 2022 to October 2023. The research team used grounded theory and conducted open, axial, and selective coding. Grounded theory is appropriate for these data given the inductive form of inquiry allows identification of new patterns and findings. Receptivity to new emergent themes was especially important since this is the first project to include farmworkers across five states, focus on multiple dimensions of farmworkers’ daily lives, and triangulate farmworker and local expert interviews.

The first author coded the deidentified data and met regularly with team members throughout the codebook development to discuss organization and interpretation of emerging themes, thereby ensuring interviewees’ intended meanings were best represented. The codebook themes that emerged during analysis for the farmworker interviews were used as categories for data coding of the local expert interviews so that local experts’ insight triangulated and deepened understanding of farmworkers’ lived experiences. This qualitative project used multiple techniques to ensure rigorous qualitative research. These were: (1) continuously applying researcher reflexivity, (2) purposive sampling, and (3) investigator and data triangulation (19).

## 3 Results

Farmworkers reported that during the pandemic access to health services varied, personal protective equipment was widely distributed, vaccine uptake was driven by multiple factors, mental and emotional well-being was challenged, and several farmworker participants contracted COVID-19. The pandemic affected family unity and rearing of children, among other factors. Lastly, COVID-19 gave rise to new job struggles, intentional changes in work practices, and minimal to no changes in the workplace.

### 3.1 Experiences with healthcare and personal health

#### 3.1.1 Access to health services

Farmworkers expressed having both ample and limited access to health services during the pandemic. The range of farmworkers’ reported access to health services reveals nuances in the impact COVID-19 had on their healthcare experiences. For instance, one dairy farmworker shared that he had employer-provided health insurance during COVID-19 and therefore could go to the hospital if necessary. Some farmworkers mentioned that there were several accessible clinics near them, that clinic staff were bilingual and respectful, and that they could easily obtain doctor appointments. A farmworker in North Carolina commented that she did not struggle at all to see a doctor during the COVID-19 pandemic and that she had been able to see the doctor every 3 months:

*“I’m going to my appointments every three months, so I never had problems with not being attended to or not having appointments…I have always, thank God, I have been able to see doctors all the time.”*

*-North Carolina Farmworker, Female*

This farmworker’s ease of healthcare access does not reflect the experiences of all farmworkers in North Carolina since it is in the context of Sampson County, which is one of 100 agricultural counties in North Carolina (20). A local expert in Washington noted that COVID-19 caused farmworkers to become more aware of the health services available in their community. Local experts also added that the following efforts enhanced healthcare access for farmworkers during the pandemic: clinic transportation provided by community health workers or farmworker employers, mobile clinics that operated in the evenings and on weekends, and community health workers visiting farmworker housing sites.

*“If the farmer [employer] will allow me to bring the mobile unit on with a provider, we do dental and medical… You know that it’ll take three hours to get through everybody…but we have weekends. We’re open on Saturday and Sunday and evenings till 9:00, so they have great access to medical and dental services.”*

*-New Jersey Local Expert, Female.*

Conversely, other farmworkers cited limited access to transportation, clinics’ narrow hours of operation, and mandatory vaccination status as healthcare barriers during quarantine. For example, a farmworker in New Jersey explained, “*Because I was not vaccinated - I did not want to get vaccinated - the doctors would not accept me in the office.”* Local experts also noted that language and lack of open doctor appointments due to high demand also restricted healthcare access for farmworkers. As stated by a local expert in Colorado, “*They [certain counties in Colorado] just had less services available for people who spoke Spanish or other languages*.”

#### 3.1.2 Access to personal protective equipment

Since farmworkers could not work from home during the pandemic, local experts spoke at length about their efforts to ensure farmworkers’ risk of infection at work was mitigated by access to personal protective equipment (PPE). Local experts organized health fairs to distribute PPE to farmworkers, visited farmworker work and housing sites to distribute PPE, and connected farmworkers with community health workers who delivered PPE and groceries. A local expert in Colorado described that, “*when we do outreach and provide it [PPE] to workers… it’s clear to me they are pretty well saturated with masks and hand sanitizer and gloves, for the most part*.”

#### 3.1.3 Vaccine uptake

Farmworkers had both positive and negative attitudes toward the vaccine. Some farmworkers expressed not wanting to get vaccinated due to apathy, religious beliefs, or skepticism toward the vaccine’s efficacy. Regarding conflict with religious beliefs, a North Carolina farmworker shared that he heard that the vaccine was the “*mark of the beast*” meaning the condemning symbol of the antichrist referenced in the Bible. Furthermore, a local expert in Georgia noted that college students from farmworker families wanted to, “*follow their parent’s wishes of not getting vaccinated*.” Therefore, family approval affected vaccine uptake.

Negative attitudes toward the vaccine were also rooted in fear. Farmworkers feared that the vaccine would kill them, cause side-effects that would require them to miss days of work, or allow the government to control them. One farmworker cited that her fear of needles also kept her from getting vaccinated. Additionally, several local experts pointed out that farmworkers feared their undocumented immigrant status would be discovered since certain vaccination sites required showing state identification or health insurance. According to local experts, many farmworkers distrust government intentions and feared the vaccine contained a chip that would track undocumented persons.

Farmworkers who received the vaccine and allowed their children to get vaccinated reported that they were motivated by a sense of familial responsibility, trust in the individuals promoting the vaccine, previous experience contracting COVID-19, desire to be able to participate in society, and belief in the vaccine’s efficacy. Farmworkers with an H-2A visa were required to obtain vaccination from November 2021 through May 2023 (21). Several farmworkers commented that conversing with other farmworkers who received the vaccine and did not experience side-effects increased their confidence in the vaccine. Examples of motivations for vaccination are featured below:

*“Well, I think that if authorities and doctors were recommending it [the vaccine] and I am one of those people who say that the government is not going to start killing people just for fun….”*

*-North Carolina Farmworker, Female.*

*“I do not know how it is where you are from, but here if the children are not vaccinated and if they do not have all the vaccines on their vaccination card, they do not let them start their school year.”*

*-Washington Farmworker, Male.*

*“I have seen that people who have been vaccinated are here and they are fine and I have been living with them…So that’s what changed my way of thinking that the vaccine is for our good.”*

*-Colorado Farmworker, Female.*

Local experts observed that community health workers were essential in promoting vaccine uptake among farmworkers because they had a relationship with the community and communicated in plain language in Spanish. A local expert in Georgia noticed that use of incentives also helped attract farmworkers to vaccination events stating, “*they [nonprofits] would offer gift cards and things like that to get people to show up.”*

Farmworkers who obtained the vaccine felt satisfied with their experience either because they found the vaccine to be easy to acquire, they had no side-effects, or they perceived it provided them with extra protection and peace of mind.

*“They did not charge us a single dime nor did anyone force us to, it was on volunteer basis, whoever wanted to could get it [the vaccine] without any difficulty. I am grateful that they vaccinated me and it worked and thank God I am alive thanks to the vaccine.”*

*-North Carolina Farmworker, Male*

*“I’ve heard that it [the vaccine] does not cure you, but it is like a defense…well what a joy because we need something like that and well I thought it was very good… And I do feel satisfied because I got it and I never, never got sick and well I felt very good about that, very happy.”*

*-Colorado Farmworker, Female*

Local experts’ efforts to ensure vaccine access included dissemination of information via Spanish radio, Head Start, Women, Infants, and Children (WIC) offices, and Facebook Live streaming events. Local experts designed vaccine promotion flyers with more pictures than words to simplify messaging. Local experts helped farmworkers register for the vaccine and partnered with local health professionals to organize vaccine drives on farms. Based on local expert interviews, farmworkers’ vaccination barriers included transportation, language barriers, illiteracy, and unfamiliarity with where the vaccine was offered. One local expert in Georgia pointed out that migratory farmworkers sometimes did not get vaccinated because they were unsure if they could receive their second vaccine dose if they had to move elsewhere. Another local expert in Georgia noted that certain organizations refused to do COVID-19 outreach to farmworkers “*because it was too political*” and that organizations that did want to help sometimes lacked the proper equipment for vaccine distribution (e.g., refrigerators).

#### 3.1.4 Personal health: mental and emotional well-being

Farmworkers reported that the COVID-19 pandemic negatively affected their mental and emotional well-being. Farmworkers felt they were living in constant fear, stress, and isolation during the pandemic. Since farmworkers would leave their home for work, they were perpetually afraid of bringing the virus home to their family or friends. Some farmworkers stated that fear has lingered in their lives and that they continue to avoid being near people, unsure if another pandemic will ensue or the vaccine will prove to be ineffective. Some farmworkers were so afraid of contracting COVID-19 that they left their job and have not returned. One farmworker in North Carolina reflected that their family was, “*living with fear each day*,” and that, “*COVID-19 caused a complete disaster by intimidating our whole family*.” A local expert in Washington observed that many farmworkers are exhausted from constant fear, “*having that big stress of having to go to work where you can potentially be exposed to COVID-19…like everyone is kind of feeling burned out now*.” This local expert also mentioned that fear of the virus kept migratory farmworkers from relocating and following their typical harvest season migration patterns.

Farmworkers also communicated experiencing great stress during the pandemic. They were concerned for nuclear and extended family members living abroad in their home countries and were anguished that they could not support them more tangibly:

“*Well, it was very, very intense, no? There was a lot of pressure here since we are alone here and the same pressure [for family] in Mexico…that was very hard…because you feel bad here and there [in Mexico], your family is suffering…I could not do anything. I could only call and call. But it feels ugly to be like this, to be here and to be talking to them over there. ‘How are you? Do not go out. Take care of yourselves*.’”

*-North Carolina Farmworker, Male.*

Caring for sick family members, socializing less, remembering to carry out each precautionary measure, scarcity of products at the store, and finances were additional stress catalysts farmworkers listed. Local experts added that several farmworker families got evicted because of financial strain and barriers faced for rental assistance.

Additionally, farmworkers’ mental and emotional well-being was affected by isolation. A New Jersey farmworker expressed that COVID-19 mitigation protocols hampered comradery at the worksite since workers could not eat lunch together and always had to be socially distanced. Regarding quarantine, certain employers rented trailers to forcibly isolate farmworkers who contracted COVID-19. A farmworker in North Carolina explained that he and his comrades did not have, “*money to survive 40 days isolated… they were afraid to die here…unable to work and make money, unable to eat well, locked up and hungry*.”

#### 3.1.5 Personal health: infection with COVID-19

Six of the 19 farmworkers interviewed contracted COVID-19. Two of these six farmworkers described their virus symptoms as minimal since they had been vaccinated before infection. A female farmworker from New Jersey reasoned that “*at one moment it [COVID-19] hit me and my family…but we did not experience grave symptoms due to our situation of already being vaccinated*.” Farmworkers who reported contracting COVID-19 mentioned avoiding the hospital (since they perceived that it may expose them to a more severe strain) and employing herbal medicines to recuperate. One of the six farmworkers reported having long COVID. Local experts shared that farmworkers were at high risk of infection due to living in multiple-person households and having to work in person. A local expert in Washington detailed that many farmworkers struggled to understand the notion of asymptomatic infection and the need to quarantine even if they did not feel ill.

### 3.2 Family life

#### 3.2.1 Family unity

Farmworkers noticed that the COVID-19 pandemic affected family unity by reducing family socialization and communication. Inability to travel to see family within the U. S. or abroad and partake in gatherings caused farmworkers to feel more disconnected from loved ones.

*“Well, the only thing that was stressful is that you could not see your family because you have older adult people, so we did not visit to try to avoid any contagion…since these were the recommendations, we tried to follow them, but it is not humane.”*

*-Washington Farmworker, Female*

*“I come from a very close-knit family where every weekend there was a meeting to see how the family is… Then the pandemic begins and the meetings end we split up and the pandemic dispersed us.”*

*-New Jersey Farmworker, Male*

Farmworkers also expressed that the pandemic minimized the frequency and quality of conversations within the family. A farmworker in New Jersey observed that during the beginning of the pandemic, conversations revolved around COVID-19 experiences but that as time passed, the quality of conversations improved:

*“…there was no communication. Yes everyone was traumatized, they were afraid of the virus…well, since this [the virus] has gone down, thank God, conversation has improved.”*

*-New Jersey Farmworker, Male*

#### 3.2.2 Difficulties with children

Farmworker mothers discussed extensively the difficulties of explaining to their children that they could not play in the park, go to stores, or engage in social activities. Mothers could tell that their children struggled to understand precautionary measures and that children were very saddened that their lives had changed. A farmworker in Washington stated that, “*not being able to take the children anywhere was the most difficult [most difficult part of the pandemic]*.” A local expert in Washington added that the impact of isolation on mental health led to increased substance and drug abuse among farmworkers’ children. Another local expert in Georgia observed that children of farmworkers only took COVID-19 precautions seriously if someone in their family died from infection.

Children’s online education was another point of tension for farmworker parents. Some farmworker mothers ceased working to supervise children’s virtual education. This loss of income further stressed limited finances in farmworker families. A farmworker in Washington appreciated that his children’s school provided the option for in-person instruction for a few hours each day. Other farmworker parents were concerned that their children did not have the opportunity to fully develop psychologically due to virtual schooling. They pointed out that online school frustrated their children and made them moody. Local experts commented that older siblings or neighbors were sometimes left in charge of farmworker children’s education, leading to subpar academic performance. Chromebook availability and limited internet access in rural areas also negatively impacted learning for farmworker children. A local expert in Washington noted that, “*those places where our [migrant] families go and find work, the internet service is really, really limited. There are only a few carriers that have coverage in that area*.”

#### 3.2.3 Long-term effects

Lastly, farmworkers identified loss of family members and close friends, greater appreciation for family, and hesitancy to leave one’s home as long-term COVID-19 consequences for their families:

*“We could not be with her [mother-in-law]… she had to die alone in the hospital… it was difficult because her decision was that she wanted us to send her to Mexico, to our country, and we could not fulfill her wish because everything was closed, the consulate was closed, everything was closed, so we could not even be with her [when she died] and we could not even fulfill her wish to bury her in her land.”*

*-North Carolina Farmworker, Female*

*“…one becomes more unified when it comes to the family. One values things like that more.”*

*-North Carolina Farmworker, Male*

*“Well, it’s like I was already used to the fact that we were in the house, well sometimes now I’m the one who then asks them [children] that if they want to go out, but we just go for a while and then we come back and now I’m the one who does not want to go out anymore, because I got used to being in the house and now I’m the one who thinks ‘but why go out?’ and ‘we can be here.’”*

*-North Carolina Farmworker, Female*

### 3.3 Workplace dynamics

#### 3.3.1 Job struggles due to COVID-19

Farmworkers cited higher workload, job loss, and shortage of employment opportunities as work-related struggles caused by the pandemic. Regarding higher workload, farmworkers reported having to work longer hours, with no salary increase, to compensate for labor shortage due to infected coworkers. A farmworker in Colorado explained that toward the end of the pandemic, farmworkers had to work long hours to meet employers’ expectations of pre-pandemic harvests despite fewer crops having been planted during COVID-19. She stated that “*it was very difficult because…there were very few vegetables and the person in charge demanded that we had to fulfill the order.*” A local expert noted that COVID-19 caused fewer H-2A workers to come to their community, creating a labor shortage that led employers to share farmworkers and take turns utilizing the same workers.

Several farmworkers mentioned that job loss was a struggle during COVID-19 because employers would replace quarantined farmworkers. A farmworker in Georgia was fired simply for being suspected to have the virus. Farmworkers felt that decreased market demand led to hour shortages and that returning to the farm after recuperating from COVID-19 was often met by opposition from co-workers who feared becoming infected. Conversely, two farmworkers stated that their employment hours were not reduced during the pandemic and that it was a good season since everyone had to keep eating. Local experts pointed out that fear of job loss due to infection caused many farmworkers to avoid COVID-19 testing or contact tracing programs. According to local experts, pandemic regulations and inflation caused some employers to close their farms, shorten employment hours, or terminate some of their farmworkers’ employment. Farmworkers described that other opportunities for additional income, such as restaurant work and construction, were also limited:

“*We did not pay him [the landlord], in total, 3 months of rent, because it was very difficult to find any other job, because restaurants actually closed. Stores closed, some stores and restaurants even went bankrupt and did not reopen and well no, there was not much to do*.”

*-Colorado Farmworker, Male*

#### 3.3.2 Helpful changes in the workplace

Farmworkers shared that some employers did and others did not take intentional steps to limit COVID-19 breakouts in the workplace. Farmworkers appreciated adjustments in work practices such as enforcing social distancing, reducing the size of work groups, and increasing intentionality in disinfecting lunch spaces. Several farmworkers reported that their employers provided PPE and alerted them if a colleague contracted COVID-19. Concerning transportation practices, some employers stopped providing shared work transportation, encouraged farmworkers to walk to work, or provided work transportation to limit farmworkers’ use of public transport. Certain employers routinely checked farmworkers’ temperatures, had farmworkers regularly take COVID-19 tests, or encouraged farmworkers to get tested for COVID-19. Farmworkers mentioned different forms of paid sick leave during COVID-19. Some farmworkers were paid for 10 days of sick leave or had 1 h of paid sick leave for every 40 h labored. Other farmworkers received $150 per week of quarantine for up to 40 days to cover food expenses. Farmworkers explained that employers disseminated COVID-19 mitigation protocols by displaying informational flyers near the bathroom or organizing an informal discussion meeting on the importance of minimizing virus transmission. Employers promoted vaccination by collaborating with mobile health clinics, providing transportation to vaccination sites, or giving financial incentives. A local expert in Washington commented that one employer invited medical professionals to host a question-and-answer session with their farmworkers before offering them the vaccine. Lastly, farmworkers and local experts cited provision of isolation facilities as an additional helpful COVID-19 mitigation strategy in the workplace. Employers isolated farmworkers in trailers or sometimes partnered with local hotels to isolate farmworkers when needed. Below are accounts of COVID-19 migration strategies experienced by farmworkers:

*“Yes, well it was also at work, yes they had a lot of requirements. We had to be using hand sanitizer, they gave us masks to protect ourselves. Oh, and well yes they asked us not to be close, to keep our distance.”*

*-Washington Farmworker, Female*

*“At the farm where I was, they [employers] gave it [COVID-19] a little more importance because… the people who did not have the vaccines, they [employers] took them [unvaccinated farmworkers] to get vaccinated so that there would not be any problems.”*

*-Colorado Farmworker, Female*

*“Well, when one [farmworker] came out infected, they [employers] tested all of us, and when they tested all of us and those who came out negative… they [employers] let a few days go by to see if they could get it [COVID-19 test] done again before sending them [farmworkers] to work with others employees.”*

*-New Jersey Farmworker, Male*

*“When we did our testing clinics and our vaccine clinics, they [farmworker employers] opened it up to all their [farmworker] family and loved ones. So even if they [farmworker families] did not live on the farm and they lived in town, they were coming on the farm to get their vaccines.”*

*-New Jersey Local Expert*

#### 3.3.3 Minimal to no changes in work environment

Several farmworkers reported that their employers implemented minimal to no changes in the work environment in response to COVID-19. For instance, some employers did not conduct regular COVID-19 tests. As expressed by a farmworker in Colorado, employers would suggest “*if you feel bad, just rest and come back when you feel better*.” Furthermore, farmworkers detailed that COVID-19 mitigation strategies were either not discussed by their employers or explained in English and thus incomprehensible. Local experts noticed that certain employers politicized and discouraged vaccination. One local expert in Colorado confided that employers would, “*either outright say they [farmworkers] could not have the vaccine or they [employers] would just make it so difficult so that they [farmworkers] would not be able to logistically like figure out a way to go get the vaccine*.” Many farmworkers added that use of PPE was not enforced and paid sick leave was not provided. Many local experts said they were actively educating farmworkers on their legal right to guaranteed paid sick leave although employers sometimes fired farmworkers who requested paid sick leave.

A farmworker in North Carolina disclosed that her employer did not provide sufficient access to bathroom facilities. The employer had 30 farmworkers share one porta-potty that was cleaned only once every 15 days. The farmworker confessed that many workers “*preferred to go to the mountains*” and practice open defecation than use the shared porta-potty.

Local experts pointed out that employers avoided reporting infection rates and avoided facing accountability for treatment of farmworkers by paying farmworkers under the table or threatening to terminate work contracts. A few farmworkers reasoned that the pandemic barely affected their work practices because they already worked outdoors, were distanced from other workers, and thus were at low risk. Some of these farmworkers stated their employer simply explained that the workers should take precautionary measures but no changes in work practices took place.

## 4 Discussion

This investigation aimed to characterize the lived experiences of farmworkers in the U. S. during COVID-19, particularly seeking to understand farmworker vaccine experiences, familial dynamics, and actions implemented by employers. Understanding farmworkers’ pandemic experiences is important since this information can serve as foundational evidence for development of interventions and policies that protect the wellbeing of farmworkers given another global disruption. According to this project’s results, COVID-19 primarily impacted three spheres of farmworker’s daily life: health, family, and the workplace. Of the emergent themes, particularly novel findings include identification of additional factors of farmworkers’ motivation for vaccination, farmworkers’ anguish concerning extended family, the deterioration of unity in farmworkers’ families, and identification of what workplace changes farmworkers deemed helpful. These novel findings widen understanding of how farmworker health can be promoted in the event of another global disruption. Although present recommended strategies (e.g., improving housing conditions and disseminating health information in Spanish) will contribute to ensuring optimal well-being of farmworkers long-term, this project shows that policymakers and public health professionals should also design and integrate actions that target farmworkers’ vaccine motivations, promote unity/connection within the extended and nuclear family, and incentivize employers to implement effective workplace changes that farmworkers deemed helpful.

### 4.1 Vaccine motivations

Farmworkers were motivated to obtain the vaccine due to belief in the vaccine’s efficacy, desire to participate in society, prior experience contracting COVID-19, a sense of familial responsibility, and having received efficacy reassurance from vaccinated farmworkers. Financial incentives, such as gift cards, were also identified as a source of vaccine motivation for farmworkers. Furthermore, farmworkers in this project attributed their vaccine satisfaction to encountering no side effects and obtaining extra protection and peace of mind. This knowledge, combined with efforts to address barriers to farmworkers’ vaccination (e.g., fear of deportation), can be leveraged to strategize more effective vaccination campaigns in the future (22–24).

Although researchers have published on the effectiveness of health fair vaccination events and recommendations for vaccinating farmworkers, self-reported farmworker COVID-19 vaccination motivation within the U. S. has limited documentation (24, 25). One qualitative study that reported farmworkers’ motivations for vaccination in the U. S. (26) found that, in Maryland and Delaware, farmworkers were motivated to obtain the vaccine because they wanted to participate in society (e.g., work or travel), protect their loved ones, and were reassured of vaccine efficacy from trusted sources who were already vaccinated. Our data confirm each of these motivators promote vaccine uptake among farmworkers. Our work builds upon these findings by discovering two additional motivators which promote farmworker vaccine uptake: (1) prior experience contracting COVID-19, and (2) financial incentives (e.g., gift cards).

### 4.2 Familial dynamics: anguish concerning extended family

Several farmworkers felt great anguish in being unable to tangibly help their family abroad. This sentiment was shared by other immigrants in the U. S. whose families live abroad (27). Researchers recommended that, to cope with this stress during the pandemic, immigrants ought to practice mindfulness, leverage social networks near sick family members, and use mobile apps (e.g., WhatsApp) to maintain communication with family in communities of origin (27). Given limited, unreliable, or inexistent internet access in rural areas, farmworkers often could not engage in web-based coping strategies (28). Farmworker advocates in North Carolina recognized the importance of bridging this digital divide and were active in providing computers and WiFi hotspots (29, 30). The use and efficacy of digital inclusion in reducing anxiety related to the care of family members abroad is understudied and merits further investigation.

The intense sorrow of not being able to physically help family abroad was one of several mental health challenges farmworkers experienced during the pandemic. Farmworkers also suffered depression, anxiety, and increase in substance use due to stressors such as fear of COVID-19 infection, caretaking demands, and financial concerns (15, 31–34). During COVID-19, these threats to mental and emotional well-being, along with limited access to mental health care, placed foreign-born farmworkers at high risk of suicide (35). This paper adds value to existent research on the mental health challenges farmworkers faced during COVID-19 by highlighting an issue not yet identified in other work: the anguish farmworkers felt in being unable to tangibly help their family abroad.

### 4.3 Familial dynamics: decline of family unity

Farmworkers in this investigation expressed that the frequency and quality of conversations with family members diminished during the pandemic. This phenomenon, coupled with reduced family socialization due to physical distancing, negatively influenced family unity. Regarding the reduced quality of conversations during COVID-19, farmworkers expressed that familial conversations focused primarily on COVID-19. This suggests that access to cell phones and internet connection does not guarantee meaningful conversation with loved ones. Research on how to encourage farmworkers to engage in deeper conversations may be valuable in the event of another mass quarantine. With respect to reduced family socialization because of physical distancing, identifying which elements of family gatherings are effective in strengthening family unity and transferable to a virtual modality may also be beneficial.

The decline of family unity among farmworker families during COVID-19 is corroborated by a national survey which reported that 1 in 4 Americans felt less close to family members toward the end of the pandemic (36). Decrease in social connection is concerning since it is adversely associated with health and well-being (37). For instance, a UK study discovered that individuals with less social connection had weaker antibody responses to the COVID-19 vaccine (38). Furthermore, a study in California found that mothers and young adults in farmworker families cited quality time with family as a stress coping strategy during the pandemic (31). Therefore, long-term actions to promote family unity among farmworkers are important for physical and mental wellbeing. This paper adds value to existent literature on social connection and farmworker familial dynamics during COVID-19 by revealing: (1) family unity declined among farmworker families during COVID-19, and (2) reduced family socialization coupled with diminished frequency and quality of conversation undermined farmworker family unity during COVID-19.

### 4.4 Helpful workplace changes implemented by employers

Lastly, regarding workplace dynamics, there is ample literature on how work practices, and lack of changes in practices, fostered poor working conditions that placed farmworkers at increased risk of infection (14, 15, 28, 39–41). For instance, employers of youth farmworkers in North Carolina did not enforce mask wearing (40). Some farmworker employers also avoided testing workers for COVID-19 due to concerns regarding the financial impact of work stoppage (28).

Less discussed, but important to recognize and replicate, are the changes in work practices that farmworkers identified as helpful. According to this data collection project, such helpful changes in work practices include employers providing transportation to vaccination sites, provision of paid sick leave, and employers regularly checking farmworkers’ temperatures, among other preventive workplace measures aforementioned in the results subsection of workplace dynamics.

Intentional collaboration between employers and health outreach workers to implement infection mitigation is crucial in promoting optimal farmworker health. Development of a positive reinforcement system that rewards farmworker employers for implementing helpful workplace changes during disease outbreaks could prove life-saving for farmworkers and beneficial to the economy.

These findings, specific actionable workplace changes that farmworkers deemed beneficial, complement the literature on employer workplace practices that did not benefit farmworker health during COVID-19. Furthermore, an analysis conducted by healthcare providers in Iowa revealed that transporting farmworkers via a cohorting process decreased COVID-19 transmission (42). Therefore, our finding that employer-provided transportation to work sites is a work practice that was deemed advantageous by farmworkers aligns with another study and strengthens the conclusion that this is an effective practice.

### 4.5 Limitations and strengths

Generalizability is often a caveat in qualitative studies due to focus on a specific subpopulation and area. In this investigation, diverse farmworkers and local experts from selected counties in five states across the U. S. were interviewed. Therefore, the results generated have potential for generalizability, although farmworkers in other states may have had different experiences than those in this sample. Nonetheless, this is the first multistate qualitative analysis on the lived experiences of farmworkers during COVID-19 (14, 41, 43, 44).

The present analysis has several strengths. First, although the farmworker industry is male-dominated, this project included insight from both female and male farmworkers. Second, this analysis triangulated information from both farmworkers and local experts to enrich the trustworthiness of findings. Third, the interview guide used to collect data was refined after collection of NCFH FCCA Phase 1 data. This extra step in methodology strengthens reliability of results.

## 5 Conclusion

Pre-pandemic literature establishes that farmworkers experience health disparities due to inequalities in economic stability, neighborhood and built environment, social and community context, and healthcare quality (3, 4, 6, 7, 45). This qualitative data collection project confirms that COVID-19 further revealed and exacerbated adverse farmworker health, work, and living conditions (14, 41, 43, 44). It expands understanding of how COVID-19 affected the lived experiences of farmworkers by specifically identifying additional factors of farmworkers’ motivation for vaccination, farmworkers’ anguish concerning extended family, the deterioration of unity in farmworkers’ families, and identification of what workplace changes farmworkers deemed helpful. Therefore, these novel findings can inform the design and implementation of legislation and interventions for farmworkers geared toward promoting effective vaccine uptake, resiliency of family unity, and implementation of workplace changes that are advantageous for their health. In the event of another global disruption, such actions can ensure that farmworker well-being does not further deteriorate.

## Data Availability

The original contributions presented in the study are included in the article/Supplementary material, further inquiries can be directed to the corresponding author.
