# Supplementary material for: Lived experiences of farmworkers from five U. S. states during the COVID-19 pandemic
Source: Front Public Health. 2025 Jun 10;13:1503383. doi: 10.3389/fpubh.2025.1503383 (PMC12185419; doi:10.3389/fpubh.2025.1503383)
Supplement: Supplementary file 1 [file Data_Sheet_1.docx]

Cover Page

## General flow of the guide & interview:

- There are 7 sections in this guide
  1. Introduction *(2-3 minutes)*
  2. Farmworker population
  3. **COVID-19 Vaccination and Services Access***
  4. Farmworker COVID-19 Experiences
  5. Emerging or current issues
  6. If time
  7. Wrap-up questions *(2-3 minutes)*
- ***Experiences with COVID-19 vaccines** section is the priority section. Make sure that all questions are asked
- “If Time” section – if you have extra time, you can use your discretion to determine how many of these questions to ask, depending on how much time is remaining, topics that have not yet been addressed, and/or topics you’d like to discuss further.
- The interview should take approximately 1 hour, however plan for 2 hours per interview. If your participant is talkative & wants to keep talking, do not cut off the interview.

## 4 Types of Questions

- **Main question:** The main questions are numbered. They will often include follow ups or probes.
- **Follow ups:** Should be asked, but can skip if 1) you are running out of time, or 2) you got the information from the original question

1. Can you tell me about your experience at work since the start of COVID-19 pandemic?

**Follow up:**

- 1. How has it changed since the beginning of the pandemic?
- **Contingent follow ups:** Should be asked if a participant responds in a certain way.

2. Have you experienced any major challenges or concerns about dealing with COVID-19 at work?

**[If yes] Follow up:**

- 1. What are they?

**[If no] Follow up:**

1. Why do you think your work has been unaffected by COVID-19?

3. Will you tell me why you haven’t received the vaccine yet?

**{*Probes - If they mention TRANSPORTATION, CULTURE/LANGUAGE, or TIME as barriers*}**

- - 1. *Can you say more about that?*
    2. *Can you give me an example?*
- **Probes:** Suggested questions to encourage the participant to give more detail, and provide an opportunity for them to answer the original question. Ask probes at your discretion, if you need more detail (they are not required)

4. Can you tell me about how the pandemic has affected your daily life?

*Probes:*

1. *What was that like in the beginning of the pandemic?*
2. *What is it like now?*

Local Expert Interview Guide

**INTRODUCTION & PURPOSE**

Hi, I'm ______ from the National Center for Farmworker Health, and we are working with several organizations that collaborate with and serve farmworkers to conduct interviews in your area. The purpose is to get information directly from community organizations or leaders serving farmworkers about the impact of COVID-19 on their lives. We hope that this data will be useful for Organizations and public health entities to advocate for and provide services for farmworkers.

To get information about the conditions workers face during this pandemic, we want to talk to a group of people who have worked closely with the farmworker community since from March 15, 2020 to now. Have you worked closely with the farmworker community since that date?

**[if yes – continue]**

**[if no – participant is not eligible]** Sorry you are not eligible to participate. Thank you for your time

Before we get started, I’ll go over more details about your participation and privacy, and then ask for your consent.

**PROCEDURES TO BE FOLLOWED**

The interview will last approximately 45 minutes to 1 hour.

**RISKS**

There is very little risk to you in answering the interview questions. If a question causes discomfort, you are free to not answer it.

**PRIVACY**

Your answers to the interview are private, and your name will not be identified in the results of this project. The record of your responses will be kept in a secure location, and only those working on the survey/project will be allowed to see it. We have asked for your name and phone number to help us schedule and conduct the interview and to compensate you for your time. We will delete your name and phone number from our records when we no longer need to communicate with you.

**VOLUNTARY PARTICIPATION & COMPENSATION**

Participating in this interview is your choice and you can refuse to answer any of the questions. You will be given $100 by check or money order. This money is for the time you are spending in this interview.

**WHO TO CALL WITH QUESTIONS**

You may call Nic Mandujano at the National Center for Farmworker Health at [Removed for Privacy] if you have questions later about the interview.

Do you have any questions?

**CONSENT**

Do you consent to participate in this interview?

- **[if yes]** Thank you for participating. Please state your name and today’s date.
- **[if no]** Thank you for your time.

I would like to record our interview to help with writing up my notes. Do I have your permission to record?

- **[if yes - START RECORDING]**

# I. Introduction

***[1-2 Minutes]***

1. Can you tell me about the work that you do with farmworkers?

# II. Farmworker Population

1. Can you tell me about the farmworkers in your community or that your organization serves?

*Probes: [if the following are mentioned, ask for more details (if not already given)]*

- - 1. *Can you tell me more about Indigenous Workers in your community?*
    2. *Can you tell me more about H-2A workers in your community?*

**Follow up:**

- 1. Have the demographics of farmworkers changed over the last 5 years?

1. Can you tell me about any migration patterns of farmworkers in the area?

*Probes:*

- - 1. *What can you tell me about Interstate migration*
    2. *Movement from other countries to U.S. for Agriculture work*
    3. *When do they arrive and from where?*
    4. *When do they leave and to where?*

**Follow up:**

1. What changes have you observed in the migration patterns of farmworkers?

*Probes:*

1. *During the pandemic?*
2. *in the last 10 years?*

# III. COVID-19 Vaccination and Services Access

1. Will you please talk about the efforts in this community to get farmworkers vaccinated?
2. Can you explain how farmworkers can access vaccines?

**Follow up:**

1. Are there any challenges that farmworkers face when trying to access vaccines?
2. If so, what are they?
3. Are there any challenges specific to Indigenous farmworkers?
4. If so, what are they?
5. Are there any challenges specific to migratory farmworkers?
   - 1. If so, what are they?
6. How would an H-2A farmworker access vaccines?
7. Other groups? **[Interviewer should ask about any other specific groups of farmworkers that may have come up in the participant’s answer to question #2]**
8. What efforts have been helpful for farmworkers to get vaccinated?

**Follow up:**

1. Have there been any strategies or efforts that have been unhelpful?
   - 1. How so?
2. Have there been any gaps in efforts to help farmworkers vaccinated?
3. Can you expand on those?
4. What other COVID-19 services do farmworkers have access to in your community?

*Probes: testing, quarantine housing, PPE, masks, etc.*

**Follow Up:**

- 1. Do farmworkers have challenges in accessing those services?

**[If yes]**

- - 1. What types of challenges exist?

**[If no]**

- - 1. What do you think makes them successful?

# IV. Farmworker COVID-19 Experiences

1. What kind of impact has the pandemic had on this farmworker community?

**Follow up:**

- 1. What kind of impact has there been on farmworkers’ daily lives?

*Probes [if top three topics are mentioned; probe for additional details “can you tell me more about, etc.: if participant does not offer a detailed response for i – iii; move to other probes]*

- - 1. ***Transportation (how the transportation experience has changed)***
    2. ***Stress***
    3. ***Family dynamics***
    4. *Housing*
    5. *Budget (change in income)*
    6. *Ability to travel (considering state restrictions & country restrictions)*
    7. *Childcare needs*
  1. How has the pandemic impacted farmworker’s employment?

*Probes:*

- - 1. *How has the pandemic impacted working conditions and work environment? (sanitation, safety measures)*
    2. *How has the pandemic impacted employment opportunities for farmworkers?*

# V. Emerging or Current Issues

1. What are the critical issues farmworkers are facing in your community right now?
2. What opportunities/solutions do you think would improve timely access to COVID-19 preventive and health care services for farmworkers in your community?
3. Is there anything else you’d like to share about farmworkers’ experiences during the pandemic?
   1. **[if the participant DOES NOT talk about something that farmworkers are currently experiencing]** Is there anything that farmworkers are currently experiencing due to the pandemic that we haven’t yet discussed?

# VI. If Time

1. How would you describe farmworker access to general health care services?

**Follow Up:**

- 1. What is needed to improve health care access for farmworkers?
  2. What has changed since the pandemic?
     1. What do you think we need to do to improve health care access during the pandemic?

# VII. Wrap Up

***[INTERVIEWER: We will end with a few quick questions]***

1. What types of crops and/or livestock do the farmworkers work in?
2. How long have you been working with farmworkers?
3. What cities/counties does your organization serve or do you work in?
4. Do you know any community leaders who are farmworkers (for example, a farmworker who has a formal or informal leadership role) who would be interested in participating in this type of interview?

**[if yes]**

**Name:**

**Phone number:**

***[INTERVIEWER: That was the last question of this interview. Your experiences are so important and we appreciate you sharing with us. Thank you for participating. Can I get your contact information so I can send your $100 payment?]***

Are you able to receive a check through mail?

**[If yes]**

Name:

Address/PO Box:

City:

State:

Postal Code:

In case we have any issues sending your payment, can I get your phone number?

Phone number:

Is that a Whatsapp number? yes / no

**[If no]** Ok, we can send a Western Union payment instead.

Name (exactly as it appears on your ID card):

City:

State:

***[INTERVIEWER: Once the transfer is made, we will follow up with a tracking number. You will need the tracking number to pick up the payment. It will be available immediately]***

In order to provide you the tracking number for your payment, can I get your phone number?

Phone number:

Is that a Whatsapp number? yes / no

***[INTERVIEWER: You should receive the check or money order tracking number in about two weeks. If not, please contact us. Thank you again.]***

**De-briefing Guide**

**Participant (context):**

What point of view does this participant have? What Info. was specific to them?

Demeanor, any changes throughout interview (list question number)

**Environment:**

Summary of experience throughout pandemic.

Strong barriers, challenges, unmet needs.

Strong facilitators, met needs, strengths.

**Storytelling/Enriching Data:**

What story from this interview is the most impactful upon review?

Which quote is the most unique/powerful?

Which quote(s) remind you of past sessions?

**Key Themes:**

General Key Themes (Circle or bold key theme that is appropriate, justify with bullet point of quote/paraphrase from interview. Note: not all themes will be represented in every interview.):

Exacerbation of previous issues due to COVID-19

Language/cultural barriers

Transportation Access

Increased Access to care

Decreased access to care

Employer Control

Relationships & Family

Changes to demographic of farmworkers

Indigenous workers experiences

Working Conditions

Mental Health

Other Key theme (write in, justify):

**Retrospective:**

Anything to note about this interview: (call drop, quality issues, etc)

Any difficult questions?

Any exceptionally useful practices?

Any updates should be made to the guide?
