# Supplementary material for: Lived experiences of farmworkers from five U. S. states during the COVID-19 pandemic
Source: Front Public Health. 2025 Jun 10;13:1503383. doi: 10.3389/fpubh.2025.1503383 (PMC12185419; doi:10.3389/fpubh.2025.1503383)
Supplement: Supplementary file 2 [file Data_Sheet_2.docx]

# Farmworker Recruitment

*Continued from survey: Thank you again for participating in our survey. Would you be interested in participating in a longer interview? It takes about 1 hour to complete and you will be compensated $100 for your time. We would like to discuss in more detail your experiences during the pandemic, including what you experienced at work, in your daily life, and with the Covid vaccines.*

*Are you available now?*

***[if yes – proceed with the interview]***

***[if no]***

*When is a good time?*

*Can I call you?*

*What is your phone number?*

*Is that a WhatsApp number?*

***[if participant wants to meet]***

*Where can we meet? (make sure you & a partner can meet in a safe location. Preferably somewhere in public during day time)*

| **Name** | **Phone number** | **WhatsApp number? y/n** | **Date & time of interview** | **Meeting location** |
| --- | --- | --- | --- | --- |
|  |  |  |  |  |
|  |  |  |  |  |
|  |  |  |  |  |
|  |  |  |  |  |
|  |  |  |  |  |
|  |  |  |  |  |
|  |  |  |  |  |
|  |  |  |  |  |
|  |  |  |  |  |
|  |  |  |  |  |
|  |  |  |  |  |
|  |  |  |  |  |
|  |  |  |  |  |

Cover Page

## General flow of the guide & interview:

- Begin with FCCA introduction & getting consent
- There are 7 sections of questions
  - Introduction questions *(2-3 minutes)*
  - Experiences during the pandemic at work
  - **Experiences with COVID-19 vaccines***
  - Experiences during the pandemic in daily life
  - Emerging or current issues
  - If time
  - Wrap-up questions *(2-3 minutes)*
- ***Experiences with COVID-19 vaccines** section is the priority section. Make sure that all questions are asked
- “If Time” section – if you have extra time, you can use your discretion to determine how many of these questions to ask, depending on how much time is remaining, topics that have not yet been addressed, and/or topics you’d like to discuss further.
- The interview should take approximately 1 hour, however plan for 2 hours per interview. If your participant is talkative & wants to keep talking, do not cut off the interview.

## 4 Types of Questions

- **Main question:** The main questions are numbered. They will often include follow ups or probes.
- **Follow ups:** Should be asked, but can skip if 1) you are running out of time, or 2) you got the information from the main question

1. Can you tell me about your experience at work since the start of COVID-19 pandemic?

**Follow up:**

- 1. How has it changed since the beginning of the pandemic?
- **Contingent follow ups:** Should be asked if a participant responds in a certain way.

2. Have you experienced any major challenges or concerns about dealing with COVID-19 at work?

**[If yes] Follow up:**

- 1. What are they?

**[If no] Follow up:**

1. Why do you think your work has been unaffected by COVID-19?

3. Will you tell me why you haven’t received the vaccine yet?

**{*Probes - If they mention TRANSPORTATION, CULTURE/LANGUAGE, or TIME as barriers*}**

- - 1. *Can you say more about that?*
    2. *Can you give me an example?*
- **Probes:** Suggested questions to encourage the participant to give more detail, and provide an opportunity for them to answer the original question. Ask probes at your discretion, if you need more detail (they are not required)

4. Can you tell me about how the pandemic has affected your daily life?

*Probes:*

1. *What was that like in the beginning of the pandemic?*
2. *What is it like now?*

**In Depth Interview Guide for Farmworkers**

**Version 3.7.2022**

**INTRODUCTION & PURPOSE**

Hi, I'm ______ from the National Center for Farmworker Health, and we are working with several organizations that collaborate with and serve farmworkers to conduct interviews in your area. The purpose is to get information directly from farmworkers about the impact of COVID-19 on their lives. We hope that this data will be useful for Organizations and public health entities to advocate for and provide services for farmworkers.

To get information about the conditions workers face during this pandemic, we want to talk to a group of people who have worked in agriculture, on animal farms, or in packing houses since from March 15, 2020 to now. Have you done agricultural work since that date?

**[if yes – continue]**

**[if no – participant is not eligible]** Sorry you are not eligible to participate. Thank you for your time

Before we get started, I’ll go over more details about your participation and privacy, and then ask for your consent.

**PROCEDURES TO BE FOLLOWED**

The interview will last approximately 45 minutes to 1 hour.

**RISKS**

There is very little risk to you in answering the interview questions. If a question causes discomfort, you are free to not answer it.

**PRIVACY**

Your answers to the interview are private, and your name will not be identified in the results of this project. The record of your responses will be kept in a secure location, and only those working on the survey/project will be allowed to see it. We have asked for your name and phone number to help us schedule and conduct the interview and to compensate you for your time. We will delete your name and phone number from our records when we no longer need to communicate with you.

**VOLUNTARY PARTICIPATION & COMPENSATION**

Participating in this interview is your choice and you can refuse to answer any of the questions. You will be given $100 by check or money order. This money is for the time you are spending in this interview.

**WHO TO CALL WITH QUESTIONS**

You may call Nic Mandujano at the National Center for Farmworker Health at [Removed for Privacy] if you have questions later about the interview.

Do you have any questions?

**CONSENT**

Do you consent to participate in this interview?

- **[if yes]** Thank you for participating. Please state your name and today’s date.
- **[if no]** Thank you for your time.

I would like to record our interview to help with writing up my notes. Do I have your permission to record?

- **[if yes - START RECORDING]**

# Introduction

***[INTERVIEWER: Let’s start with a few questions to get to know you…] [2-3 Minutes]***

1. Can you tell me a little bit about your work in agriculture?

*Probes:*

- - 1. *What crop or livestock do you work with?*
    2. *What task do you do?*

# Experiences During the Pandemic at Work

***[INTERVIEWER: Now we will discuss about your experiences at work.]***

1. In general, what do you think about COVID-19?

**Follow up:**

- 1. How have your feelings about COVID-19 changed over time?

1. Can you tell me about your experience at work since the start of COVID-19 pandemic?

**Follow up:**

- 1. How has it changed since the beginning of the pandemic?

1. Have you experienced any major challenges or concerns about dealing with COVID-19 at work?

**[If yes] Follow up:**

- 1. What are they?
  2. How did [insert challenge/concern] affect you or your work?

**[If no] Follow up:**

1. Why do you think your work has been unaffected by COVID-19?

*Probes:*

- - 1. *Did you experience a change in your work schedule or opportunities for work?*
    2. ***[If H-2A]:*** *How did the pandemic impact the visa (or H-2A work permit) process for you (for example, timing, recruitment, how/when you entered the U.S.)?*

1. Did your employer change their operations to protect workers from COVID-19?

**Follow up:**

- 1. **[if yes]** What do you think of the changes that were made?
  2. Why do you think that?
  3. What changes do you think needed to be made but didn’t happen?
  4. If you were in charge of the farm, what things would you have done differently?

1. What happens if you or a co-worker can’t work due to COVID related issues?

**Follow up:**

- 1. What does your employer do?

*Probes*

- - 1. *What if a worker is sick with COVID?*
    2. *What if a worker needs to take care of a loved one with COVID?*
    3. *What if a worker needs to quarantine due to being around someone else who has COVID?*

# Experiences with COVID-19 Vaccines

***[INTERVIEWER: Now we will discuss your thoughts about the COVID vaccines.]***

1. Have you gotten your COVID-19 vaccine(s) (the primary series)?

**[If yes] Follow up:**

- 1. Can you share about your experience getting the vaccine(s)?

*Probes:*

- - - 1. *Where did you get the vaccine from?*
      2. *Was there anything challenging about getting the vaccine?*
      3. *Was there anything that made getting the vaccine easy?*
  1. What made you decide to get vaccinated?
  2. Were you ever unsure about getting the vaccine?

*Probes:*

- - - 1. *If so, why?*
      2. *If not, why not?*

**[If no] Follow up:**

- 1. Do you plan to get the vaccine?

**[if yes] Follow up:**

- - 1. What helped you decide to get the vaccine?
    2. Will you tell me why you haven’t received the vaccine yet?

**{*Probes - If they mention TRANSPORTATION, CULTURE/LANGUAGE, or TIME as barriers*}**

- - 1. *Can you say more about that?*
    2. *Can you give me an example?*
    3. *Was there any other time that was an issue?*

**[if no] Follow up:**

- - 1. Will you tell me why you decided not to get the vaccine?
    2. Is there anything that would convince you to get the vaccine?

1. Has your opinion about getting vaccinated changed over the course of the pandemic?

**[If yes] Follow up:**

- 1. What led to that change?

**[INTERVIEWER - only ask Q9 if participant has received primary series]**

1. Have you received a COVID-19 booster?

**[If yes] Follow up:**

- 1. What impacted your decision to get a booster?
  2. Can you tell me about your experience getting the booster?

**[If no]** **Follow up:**

- 1. Do you plan to get the booster?

**[if yes]** **Follow up:**

- - 1. What do you need in order to get the booster?

**[if no]** **Follow up:**

- - 1. Can you tell me why you have not received a booster?
    2. Is there anything that would motivate (or convince) you to get the booster?

# Experiences During the Pandemic in Daily Life

***[INTERVIEWER: Now we will discuss what your experience was like in your daily life throughout the pandemic.]***

1. Can you tell me about how the pandemic has affected your daily life?

*Probes:*

1. *What was that like in the beginning of the pandemic?*
2. *What is it like now?*

***{Probes - If they mention TRANSPORTATION, STRESS, FAMILY, or TRAVEL RESTRICTIONS...}***

- - 1. *Can you say more about that?*
    2. *Can you give me an example?*

## V. Emerging or Current Issues

***[INTERVIEWER: Lastly, we will discuss any important current issues to you and your community.]***

1. As a farmworker, what current issues are you (or farmworkers in general) facing now?

*Probes:*

- - 1. *What current issues are farmworkers facing related to labor or working conditions?*
    2. *What current issues are farmworkers facing related to COVID?*
    3. *What current issues are farmworkers facing related to health?*

# VI. If Time

1. Many people have decided not to get vaccinated – what do you think about that?
   1. Why do you think it’s important to get vaccinated?
2. Did you ever get COVID?

**[if yes] Follow up:**

- 1. Can you tell more about that experience?

1. Throughout the pandemic, many people had difficulty seeing a doctor or getting the health care they needed. Did you experience that?

**[if yes]** **Follow up:**

- 1. Can you tell me more about your experiencing getting the health care you needed?

1. Do you have any health conditions (like diabetes or heart disease)?

**[if yes] Follow up:**

- 1. What has it been like managing your [health condition] during the pandemic?
     1. Early in the pandemic? Today?

1. Will you please talk to me about your relationship with your family since COVID started?

**Follow-up**

- 1. What kinds of conversations have you had around COVID?
  2. What (if any) changes have you made to your daily life since COVID?

1. Many farmworkers were not able to see their families that live in different states or countries. Did you experience that during the pandemic?

**[if yes] Follow up:**

- 1. Can you tell me more about that experience?

1. Can you describe what your working relationships are like?

**Follow up:**

- 1. With Farmworkers?
  2. With Crew leaders, supervisors?
  3. With Contractors, employers?
  4. Did your relationships change throughout the pandemic? How so?

# VII. Wrap-up Questions

***[INTERVIEWER: We will end with a few quick questions about yourself. About 2-3 minutes]***

1. Where were you born? (country and state)
2. Do you live here with family?
3. How do you identify racially or ethnically?
4. What languages do you speak?
5. What is your gender?
6. How long have you been working in agriculture?
7. If we have some follow-up questions in a few months, can we call you?

**[if yes]**

**Phone number:**

**Is that a Whatsapp number?:** yes / no

1. Do you know any other farmworkers who would be interested in participating in this interview?

**[if yes]**

**Name:**

**Phone number:**

***[INTERVIEWER: That was the last question of this interview. Your experiences are so important and we appreciate you sharing with us. Thank you for participating. Can I get your contact information so I can send your $100 payment?]***

Are you able to receive a check through mail?

**[If yes]**

Name:

Address/PO Box:

City:

State:

Postal Code:

In case we have any issues sending your payment, can I get your phone number?

Phone number:

Is that a Whatsapp number? yes / no

**[If no]** Ok, we can send a Western Union payment instead.

Name (exactly as it appears on your ID card):

City:

State:

***[INTERVIEWER: Once the transfer is made, we will follow up with a tracking number. You will need the tracking number to pick up the payment. It will be available immediately]***

In order to provide you the tracking number for your payment, can I get your phone number?

Phone number:

Is that a Whatsapp number? yes / no

***[INTERVIEWER: You should receive the check or money order tracking number in about two weeks. If not, please contact us. Thank you again.]***

**De-briefing Guide**

**Participant (context):**

What point of view does this participant have? What Info. was specific to them?

Demeanor, any changes throughout interview (list question number)

Common words or phrases throughout

**Environment:**

Summary of experience throughout pandemic.

Strong barriers, challenges, unmet needs.

Strong facilitators, met needs, strengths.

**Storytelling/Enriching Data:**

What story from this interview is the most impactful for the general public?

What story from this interview is the most impactful for policy makers/those with power?

Which quote is the most unique compared to previous interviews?

Which quote(s) remind you of past sessions?

**Key Themes:**

General Key Themes (Circle or bold key theme that is appropriate, justify with bullet point of quote/paraphrase from interview. Note: not all themes will be represented in every interview.):

Exacerbation of previous issues due to COVID-19

Language/cultural barriers

Transportation Access

Increased Access to care

Decreased access to care

Employer Control

Relationships & Family

Changes to demographic of farmworkers

Indigenous workers experiences

Working Conditions

Mental Health

Other Key theme (write in, justify):

**Retrospective:**

Anything to note about this interview: (call drop, quality issues, etc)

Any difficult questions?

Any exceptionally useful practices?

Any updates should be made to the guide?
